# Barriers to uptake of refractive error services in a rural community in Enugu, south-East, Nigeria

**DOI:** 10.4314/ahs.v24i4.43

**Published:** 2024-12

**Authors:** Nkiru Zuada Nwachukwu, Daniel Chukwu Nwachukwu, Boniface Ikenna Eze

**Affiliations:** 1 Department of Ophthalmology, University of Nigeria Teaching Hospital, Ituku-Ozalla, Enugu, Enugu State, Nigeria; 2 Department of Physiology, College of Medicine, University of Nigeria, Enugu Campus, Nigeria

**Keywords:** Refractive errors, barriers, rural community, black population

## Abstract

**Background:**

Uncorrected refractive error remains a leading cause of visual impairment. The utilization of refractive error care services in many Nigerian communities is poor in spite of its availability.

**Objective:**

To determine the barriers to uptake of refractive errors care services in a rural community in Enugu State, Nigeria.

**Methods:**

A descriptive, cross-sectional, population-based survey with mixed method was adopted. The study instruments were a pre-tested, researcher-administered questionnaire and focus group discussion (FGD). Quantitative data were subjected to descriptive and comparative statistics and FGD was analyzed using ATLAS.ti. P value <0.05 was considered significant.

**Results:**

A total of 522 adults comprising 307(58.8%) males and 215(41.2%) females aged 43 ± 31.6 years participated in the study. Lack of felt need 235(45.02%), affordability 200(38.31%), stigmatization 184(35.25%) and distance to health care facility 88(16.86%) were the major factors that hindered the uptake of refractive errors care services in Amorji community. Age significantly associated with lack of felt need (P=0.001) and affordability (P=0.001). Educational status significantly associated with stigmatization (P=0.002) and lack of felt need (P=0.021). Results from FGDs were in agreement with those obtained from the questionnaire-based part of the study.

**Conclusion:**

The major factors that militated against the uptake of refractive care services in the community were lack of felt need, affordability, stigmatization and distance from health care facility. Good health education, planning and implementation of eye care services programmes may improve the uptake of refractive care services in such rural community.

## Introduction

The leading causes of vision impairment and blindness at a global level are refractive errors and cataracts.^1^

Globally, at least 2.2 billion people have a near or distance vision impairment. In at least 1 billion of these, vision impairment could have been prevented or is yet to be addressed.^2^ In Nigeria, 2.46 million adults were reported to be visually impaired and uncorrected refractive error accounted for 77.9% of mild, 57.1% of moderate and 11.3% of severe visual impairment 3. Uncorrected refractive errors are the second leading cause of treatable blindness, globally^2^,^4^. It adversely impacts on the individual's social life and economic activities by restricting educational and employment opportunities of otherwise healthy individuals 4. Consequently, it leads to substantial economic losses by the individual and the society and compromises the individual's independence, well-being and overall quality of life 5,6. Refractive error commonly occurs earlier, its duration of effect is significant as they can account for higher number of blind person years compared to cataract^5^.

The impact of Vision 2020 was a heightened awareness of the problem amongst all stakeholders and the widespread acceptance of a doable district (community) level solution. The specific spin offs from this were an increased implementation of rapid assessments of avoidable blindness (RAABs), including a huge increase in the human resources development strategies most importantly human resources to address refractive error.^7^ Refractive error can be corrected with spectacles, contact lenses and refractive surgeries. These corrective measures are available, affordable and cost-effective, yet utilization has been reported to be poor^8^. In low- and middle-income countries, ignorance and stigmatization were reported to be major barriers to accessing refractive error care services^8^. The Nigerian Blindness and Visual Impairment Survey observed that 2.14 million adult Nigerians would have their vision improve from <6/12 to 6/12 or better with spectacles, yet only few have spectacles^3^. Another previous study identified factors responsible for the underutilization of refractive error care services to include poor knowledge and affordability, insufficient provision of affordable corrective lenses and poor compliance with spectacle wear caused by cultural disincentives^9^.

The health-seeking behavior of an individual is determined by a lot of factors which include socio-demographic factors, social structures, cultural beliefs and practices, gender discrimination, political and economic systems, disease pattern, health care system itself and the approaches by the awareness and knowledge of it, beliefs and attitude to it^10^,^11^. Epidemiological studies worldwide have highlighted the escalating prevalence of refractive error^4^,^12^,^13^. The burden of refractive error is set to increase in the coming years especially in low-income countries^14^.

There is paucity of information on the psychosocial effect of refractive error and possible reasons for the low uptake of refractive care services in rural communities in Nigeria. The present study is an evidence-based one which sought to identify the brriers to uptake of refractive care services in a rural community in Enugu State, South East, Nigeria. This will encourage government and non-governmental organization to develop and put measures to enhance the uptake of these services in our rural underserved communities.

## Materials and methods

Study design: The study adopted a descriptive, cross-sectional, population-based survey design with mixed method approach (quantitative and qualitative).

### Study location

The study was conducted in Amorji community, Enugu-East Local Government Area, Enugu State, South-East, Nigeria. The main occupations of the inhabitants are: farming, trading and artisanship. The community has a population of about 50,200 people and constitutes one political ward in Enugu East local government. It is made up of 14 villages which are divided into 3 administrative zones based on their ancestral history. It lies in the tropical rainforest climatic belt with two seasons of the year: Dry and Rainy seasons. The inhabitants are predominantly ethnic Igbos, with Igbo and English as their main languages.

### Inclusion criteria

1. All individuals, aged 18 years or older, who have resided continuously in Amorji community for at least 1 year prior to the commencement of the study and voluntarily gave their consent to participate in the study.
2. Those who wore spectacles for correction of refractive errors previously but stopped at least 6 months prior to the study.

### Exclusion criteria

1. Individuals with presbyopia were excluded from the study.
2. Those who refused to give their consent and those that have not lived in the community for up to 1 year prior to the study.

### Sample size

The minimum sample size was determined using the Fisher's formula.

N= Z2 Pq

d2

Where

N = desired sample size

Z = 1.96 i.e standard normal deviate at confidence interval of 95%

P = 28.9% (0.29) i.e prevalence value previously reported in a similar survey^15^.

q = 1 – P

d = desired precision due to random sampling error of 5% = 0.05


$$\begin{array}{ccc} N & = & \left(1.96\right)2\;\left(0.29\right)\;\left(1-0.29\right)\\ & & \left(0.05\right)2\\ & = & 79.098/0.0025\\ & = & 316.3 \end{array}$$

The calculated sample size of 316 was multiplied by 1.5, the design effect due to cluster sampling method, to give a new sample size of 474. To take care of refusals to participate in the study, the calculated new sample size was increased by10.0% to obtain a modified sample size of 522.

### Sampling technique

A multistage cluster random sampling technique was used to select Amorji among the 21 communities in Nike, Enugu state. The proportion of 522 participants from each zone was caculated taking into cognizance their Population, thus zone 1= 131; zone 2= 131 and zone 3=260. Using a systematic random sampling, sampling interval (k) was calculated as k = N/n, where N = number of households in the selected village; n= number of participants recruited in the selected village.

### Quantitative Study

From each of the selected household, one eligible adult was recruited by random selection. The participant was taken to a private place within his/her home, granted one-on-one interview and questionnaire administered by the principal researcher and her assistant. The selection continued until the required number of participants was achieved. Any household in which no eligible participant was around for recruitment or in which none gave voluntary informed consent was skipped without revisiting the household. A total of 5 households declined being part of the study due to religious beliefs and faith.

### Qualitative Study

A total of 6 FGDs comprising 3 all-male and 3 all-female groups, with 10 participants in each group, were conducted in the 3 zones. Participants were drawn from women, men, youth and religious societies by convenience sampling. Each focal group discussion was homogenous in age and genders to enable participants express their true opinions. The criteria for participation were 18 years or older, uninterrupted residence in the study community for the past 1 year and non-participation in the preceding questionnaire-based part of the same study. The discussion with each focus group was conducted using FGD guide and recorded^16^.

### Materials

#### 1. Questionnaire

A pre-tested structured questionnaire consisting of 3 sub-sections partly developed de novo and partly adapted from a previous study17 was used.

#### Section A

Provided information on the socio-demographic characteristics of the participants.

Section B: Provided information on barriers to uptake of refractive errors services

#### Section C

Provided information on attitude towards spectacle wear

2. Informed consent form

3. Writing materials

### Pilot study and pre-test

A pilot study was conducted in Iji-Nike, a neighbouring community to assess the feasibility of the planned survey and ascertain the construct validity and psychometric reliability of the study questionnaire. Feedbacks from the pre-test on the content, flow and interpretation of the questionnaire, were used to modify the questionnaire to achieve the desired study objectives.

### Data analysis

Data was collected, cleaned, coded and entered into a computer; a copy of the cleaned data was stored in an external hard drive. Quantitative data were analyzed using the Statistical Package for Social Sciences version 21.

Descriptive statistics was carried out to categorize participants by socio-demographic factors and barriers to uptake of refractive error services and then presented as frequencies, percentages and proportions. Bivariate comparative test for inter-group differences was performed using Chi-square for categorical and Student-t test for continuous variables. P values < 0.05 were considerd significant.

### Qualitative analysis

The notes and tape-recorded information from the FGDs were analyzed using the ATLAS.ti 8 Windows software package. Each passage of speech in the FGD was detected and coded. All the responses of each participant were identified, recollected and coded. The responses of participants from different FGDs were added to the ATLAS.ti and categorized according to age, sex, occupation, marital status and barriers to uptake of refractive error care services. Comparisons were done across different socio-demographic factors and their relationship with different barrier

## Results

The average age of the participants was 43 ± 31.6 years; their age and sex distribution are shown in Table 1. Many of the participants had no formal education 173 (33.1%); their major occupation was farming 139 (26.6%) (Table 2).

**Table 1:** Age and sex distribution of participants

Age group (Years)		Sex		n(%), N= 522
	Male		Female	
18-30	71(23.1)		49(22.8)	120(22.9)
31-40	60(19.5)		17(8)	77(14.6)
41-50	93(30.3)		75(34.9)	168(32.2)
51-60	57(18.6)		56(26)	113(21.6)
61-70	19(6.2)		13(6.0)	32(2.3)
71-80	5(1.6)		3(1.4)	8(6.1)
				
81-90	2(0.7)		2(0.9)	4(0.7)
				
Total (%)	307(58.8%)		215(41.2%)	522(100)

**Table 2:** Participants' socio-demographic characteristics

Characteristics	n(%), N=522
**Educational status**	
No Formal education	173(33.1)
Primary	148(28.4)
Secondary	140(26.8)
Tertiary	61(11.7)
**Occupation**	
Farming	139(26.6)
Trading	80(15.3)
Civil servant	76(14.6)
Artisanship	90(17.3)
Student	68(13)
Retiree	12(2.3)
Unemployed	57(10.9)
**Marital status**	
Single	203(38.9)
Married	264(50.6)
Widowed/Divorced/Separated	55(10.5)

Few participants 36(6.9%) had worn spectacles in the past and were no longer wearing them at least 6 months to the time of the study and their reasons were: Loss 8(1.5%); broken 7(1.4%) and the remaining 21(4.0%) felt it was not necessary anymore (Table 3). The majority of the participants 274 (52.5%) would not allow their children to wear spectacles and believed it would have negative psycho-social consequences on them (Table 3). Majority believed that it will make people see them as visually handicapped 244 (46.7%); many also believed it will cause their eyeball to sink 219 (42.0%). Majority of the participants 330 (63.2%) would not get married to individuals wearing eye glasses (Table 3).

**Table 3:** Attitudes towards spectacle wear

**Response n (%), n =522**	
**Have you won spectacles in the past?** Yes 36(6.9) No 486(93.1) **If yes, what was your reason for not wearing your spectacles?**	
Lost them 8(1.5)	
Got broken 7(1.4)	
Spectacles not necessary 21(4.0)	
**Would you wear spectacles if prescribed by the doctor?** Yes 354(67.8) No 168(32.2) **Would you allow your children to wear spectacles?** Yes 248(47.5) No 274(52.5) **What effect do you think spectacles have on the eye?**	
Damage	53(10.1)
Weaken	104(19.9)
Sink eyeball	219(42.0)
Improve vision	78(15.0)
Worsen vision	26(5.0)
Don't know	42(8.0)
**What is your general attitude toward individuals who wear glasses?**	
Visually handicapped	244(46.7)
Intelligent	42(8.0)
Despise	29(5.6)
Indifferent	196(37.6)
Bookworms	11(2.1)
**Would you marry anyone with refractive error?**	
Yes	192(36.8)
No	330(63.2)
**Do you think two individuals with refractive error should marry?**	
No 287(55.0)	
Yes 235(45.0)	

**Figure 1:**
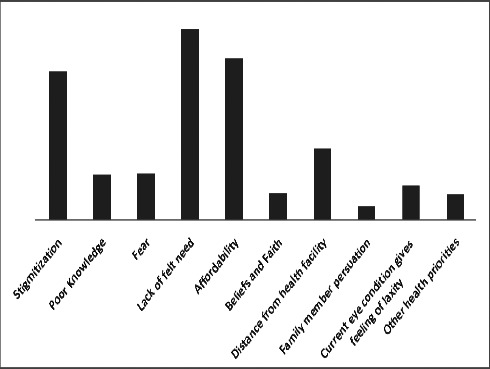
Number of participants affected by different barriers and their percentages

Lack of felt need 235 (45.02%), affordability 200 (38.31%), stigmatization 184 (35.25%) and distance from health facility 88 (16.86%) were the major barriers identified in the study community (Figure 1). Other barriers identified were fear, poor knowledge, religious beliefs and faith etc. (Figure 1).

Age significantly associated with lack of felt need and affordability (p=0.001), poor knowledge (p= 0.004) and stigmatization (P=0.01) (Table 4). Educational status significantly associated with stigmatization (P=0.002), lack of felt need (p=0.02), fear (p=0.03) and poor knowledge (p=0.04). Gender, occupation and marital status did not have significant association with any of the barriers.

The results obtained from FGD were in agreement with those from the quantitative study. Many of the participants responded to some of our questions thus:

Question: Since you are aware of refractive error, why haven't you seen a doctor to check your eyes?

Response: ‘*I can cope with my sight and do not see the need to go and spend a whole day in the hospital*’.

Question: What is your opinion about people in your village/community that wear spectacles?

Response: ‘*They are visually handicapped*’

Question: Would you marry someone wearing spectacles?

Response: ‘*No, I will not, they are many normal girls everywhere, why should I marry someone that is almost blind?*’

Question: Would you allow two individuals wearing spectacles to marry each other?

Response: ‘*They can if they want, after all love is blind*’, they said sarcastically. ‘*They should be prepared to spend most of their money on treatment*’.

Question: What is your view or opinion on the effect of spectacles on the eyeball?

Response: ‘*spectacles sink the eyeball*’.

‘*I am proof of that, can't you see my eyeball?*’ responded another participant who had worn spectacles in the past.

## Discussion

The majority of participants were males; this may be due to the fact that our study was done in a community where most of the women are petty traders in the market and farmers. Majority of these women are usually the bread winners for the families and are hardly available during the daytime. The male preponderance of the participants in this study is similar to that reported in a Pakistan community based study^18^, and that in India^8^ (though the participants were much younger). Majority of participants 349 (66.9%) attained different levels of formal education, while 173 (33.1%) had no formal education. They were mostly farmers, artisans and traders and majority was married 319(61.1%). These socio-demographic characteristics were similar to those in the Pakistan study^18^ but differed from another study done in India^19^ where most of the were university graduates and were not married.

Refractive error is a cause of visual impairment whose corrective measures are affordable but underutilized in our environment. This study identified the major barriers to uptake of refractive error services to be lack of felt need (45.02%), Affordability (38.31%), stigmatization (35.25%) and distance to health facility (16.86%). The lack of felt need was more predominant in older people (60 years and above) while stigmatization was observed more in the younger participants. Poor affordability was observed across all ages. In previous studies in India^8^, and Pakistan 18, the major factors that hindered the uptake of refractive error care services were affordability and ignorance. Though affordability was the second leading factor in the community, majority of the participants had knowledge about refractive error but do not see the need to ‘waste their time and scarce resources’ in the hospital when they can cope with their vision. Many of the participants depend on their daily income to feed their families and going to hospital for eye check wasn't seen as a priority; some felt that it would deprive them their source of income. In spite of the fact that many of the participants had some difficulty with distant vision, they felt they could cope with it. They did not see the need to seek for corrective measures since they could cope with their day-to-day tasks. This is similar to findings in other previous studies in Kenya^20^, and India^21^. The lack of felt need observed in the present study was also associated educational status; it was predominant in those with no formal education.

Most of the participants were peasant farmers, traders and artisans with relatively low income. This could be responsible for poor affordability being the second leading barrier to uptake of refractive care services in the community. Most of them felt they would rather use their little money to solve ‘important’ family problems rather than visit an eye care facility. Stigmatization was a major barrier identified in the community. Many of the participants (63.2%) rejected marriage to someone wearing spectacles and 55% disapproved marriage between two individuals wearing spectacles. Their major reason was that the children from such marriages will inherit the disease and may go blind early in life. This view is similar those expressed by respondents in two previous studies in India^8^ and Parkistan^18^ where many of them did not support marriages between two individuals with refractive error. Surprisingly, a high level of stigmatization against spectacle wear was observed in this community in spite of many of the participants having knowledge of refractive error. Many participants (including those from FGD) viewed those wearing spectacles as ‘visually handicapped’. Many (especially the female participants) believed wearing spectacles will reduce their chances of getting married; they preferred to cope with their poor vision. This fear was corroborated by the views of majority of the male participants (both in survey and FGD) who vehemently rejected marriage to girls wearing spectacles. This is similar to the findings in the Pakistani study^18^, where spectacles were described as a cosmetic blemish. Many participants also believed that their little resources will be spent on treatment and that their children will inherit the disease and possibly go blind early in life. Thus, stigmatization, fear and cultural beliefs also hindered the uptake of refractive care services in the community. A previous study also reported cultural beliefs as a major disincentive for spectacle wear^9^. Public enlightment programme in such communities will assist in reducing the level of stigmatization and create proper awareness.

Distance to a health facility was also a strong factor that militated against the uptake of refractive care services in the community. There were no eye care facilities within 10 km radius from the community. This coupled with the poor means and high cost of transportation might have contributed to the observed apathy towards uptake of refractive error care services in the community. Age significantly associated with stigmatization, affordability of refractive error services and distance to healthcare facility; the middle age groups were mostly affected. Non-formal educational status was significantly associated with stigmatization, ignorance, affordability and fear.

Identifying the barriers to utilization of refractive error care services has huge implications for the planning of eye care services. In addition to public enlightment programmes, such communities with low income should be considered in terms of affordability and distance to health facility. If an individual cannot afford to buy spectacles, most likely he will be unable to visit a health facility situated further from where he lives. The health seeking behavior of people of low income can be affected by these two factors.

## Conclusion

Lack of felt need, poor affordability, stigmatization and distance to healthcare facility were the major factors responsible for the poor uptake of refractive care services in Amorji community. Majority of those affected had no formal education. These situations can be mitigated through proper health education, planning and implementation by government and non-governmental organizations.

## Data Availability

All data or supporting data will be provided on request at any time.

## Declarations

Ethics approval and consent to participate: Ethical approval for this study was obtained from the Medical and Health Research Ethics Committee of the University of Nigeria Teaching Hospital, Ituku-Ozalla, Enugu (NHREC/05/01/2008B-FWA00002458-1RB00002323). The study was carried out in compliance with the tenets of the 1964 Helsinki Declaration, as amended in London, in 2008, on research involving human subjects.
